# Biochemical and Molecular Characterization of *Musa* sp. Cultured in Temporary Immersion Bioreactor

**DOI:** 10.3390/plants12213770

**Published:** 2023-11-04

**Authors:** Christopher A. Sambolín Pérez, Rosalinda Aybar Batista, Sullymar Morales Marrero, Dinorah Andino Santiago, Axel Reyes Colón, Juan A. Negrón Berríos, Ángel Núñez Marrero, Alok Arun

**Affiliations:** 1 Institute of Sustainable Biotechnology, Inter American University of Puerto Rico, Barranquitas, PR 00794, USA; csam1223@intersg.edu (C.A.S.P.); raybar@br.inter.edu (R.A.B.); smorales@br.inter.edu (S.M.M.); axereycol@gmail.com (A.R.C.); janegron@br.inter.edu (J.A.N.B.); anunez@br.inter.edu (Á.N.M.); 2 Department of Natural, Computational and Exact Sciences, Inter American University of Puerto Rico, Ponce, PR 00715, USA; dinorahandino00@gmail.com

**Keywords:** temporary immersion bioreactor, micropropagation, *PEPC*, *Rubisco*

## Abstract

The genus *Musa* sp. contains commercially important fleshy fruit-producing plants, including plantains and bananas, with a strong potential for providing food security and sources of revenue to farmers. Concerns with the quality of vegetative tissues along with the possibility of the transmission of phytopathogens makes the availability of healthy plantlets limited for farmers. Micropropagation of plantains offers an alternative to producing large numbers of plantlets. However, conventional methods of micropropagation have high production costs and are labor-intensive. Recently, the temporary immersion bioreactor (TIB) has emerged as an alternative to conventional micropropagation (CM) methods. Our work utilized SEM microscopy (scanning electron microscope) and molecular and biochemical tools (qRT-PCR and ICP-OES) to characterize and compare the morphological properties, elemental composition, and photosynthetic gene expression of plantains cultured on TIB. Additionally, morphological features of growth and propagation rates were analyzed to compare outputs obtained from TIB and CM. Results showed higher growth and multiplication rates for plantlets cultivated in TIB. Gene expression analysis of selected photosynthetic genes demonstrated high transcript abundance of phosphoenolpyruvate carboxylase (*PEPC*) in plantain tissues obtained by TIB. Elemental composition analysis showed higher content of iron in plantains grown in TIB, suggesting a potential correlation with *PEPC* expression. These results demonstrate that micropropagation of *Musa* sp. via the liquid medium in TIB is an efficient and low-cost approach in comparison with solid media in CM.

## 1. Introduction

*Musa* sp. is a plant genus of horticultural crops that are valuable because of their rich nutritional components, and these include bananas and plantains [1]. *Musa* sp. is native to Asian regions, and nowadays these can be found throughout other subtropical and tropical regions [2]. The genus *Musa* comprises major food crops produced all over the world, including 130 countries [3]. Although in Puerto Rico, plantain crops are the most widely produced crop representing 55.2% of total crop production, these crops were significantly impacted by hurricanes Maria and Fiona in 2017 and 2022, respectively [4]. These environmental events led to shortage of plantains in Puerto Rico, which affected the availability of fleshy fruit in the region. Identifying food insecurity factors and associated risks can help to improve programs and enhance techniques to fight food insecurity, at both the local and global levels [5]. Since plantain has a very high demand in the Caribbean region, high dependence on imported fleshy fruits have been affecting the local farmers [6]. The production and the availability of new plantlets are limited by the lack of starting plant material [7]. Another concern for farmers is the transmission of lethal pests, including viruses and fungi (for example, Black Sigatoka disease) when using conventional field-grown shoots in their farms. The limited availability of starting plant material and challenges associated with pest prevalence has increased the interest in plant micropropagation [7]. Plant micropropagation (plant tissue culture) provides an alternative solution for mass-propagation of disease-free plantlets in a short time under laboratory conditions [3]. Conventional micropropagation (CM) of *Musa* sp. using semi-solid medium demands high production costs that eventually may limit its commercial use [7]. The significance and prominence of plant biotechnological advances rely on the search for novel methods to improve crop production in plants [8]. For example, replacing solid plant tissue culture media with liquid plant tissue culture media has provided a solution for automation and decreased the production budget in commercial plant tissue culture facilities [7,8]. The temporary immersion bioreactor (TIB) allows temporary immersion of the explants in the liquid media [9]. Additionally, the positive and consistent effects of TIB on in vitro shoot regeneration have been proved for a variety of plants, with TIB thus emerging as a promising new technique for in vitro plantain propagation [7]. The TIB system promotes certain advantages that allow for the normal development of the plantlets [10]. Furthermore, TIB promotes optimal conditions for nutrient acquisition and humidity with minimum liquid contact [11]. Additionally, enhanced oxygen transport helps to develop a better gas exchange by reducing oxygen limitation. The controlled environmental conditions provided by TIB system protect the tissues, which preserves the tissue integrity and improves the morphology and physiology of the organs [12]. This method of automated propagation has provided a possible solution for reducing conventional micropropagation costs [13]. Additionally, TIB has been functional and successful in several valuable crops, such as banana [14], pineapple [10], and plantain [7]. Further, TIB-grown plants have demonstrated increased growth with significant increase in the yield [15].

Studying the developmental stages of plantain in a TIB environment is essential for evaluating plant physiology at a molecular and biochemical level. Analyzing photosynthetic gene expression may help us understand how a TIB environment can alter plant response during growth. Rubisco is a multifunctional enzyme that catalyzes the reactions of carboxylation and oxygenation. Carboxylation is the first stage in which CO_2_ fixation occurs [16,17], and is also the rate-limiting factor of photosynthesis [18]. Previous reports have suggested that the availability of the small subunits of Rubisco enzyme upregulates the transcript levels of the larger subunits of Rubisco enzyme [19]. These small subunits of Rubisco enzymes promote conformational changes in the other subunits, resulting in an improvement of the catalytic rate [19]. Additionally, on C4 plant species, the phosphoenolpyruvate carboxylase (PEPC) catalyzes the first carboxylation reaction of photosynthesis in the mesophyll cells [20]. Additionally, PEPC plays a key role in C4 photosynthesis, since it is involved in a variety of mechanisms, such as anaplerotic metabolism, stomatal opening, and pH regulation [21]. However, during micropropagation, PEPC is responsible for the mobilization of sugars through an anaplerotic route to guarantee the supply of carbon skeletons for amino acid synthesis [22]. Moreover, it has been documented that ex vitro plantains from TIB exhibit higher *PEPC* activity, and this is suggested because the TIB system mimics outdoors conditions, leading to high activity of PEPC enzymes and a higher photosynthetic rate [23]. Additionally, examining the elemental composition could reveal metal ions that potentially act as cofactors during the expression of the interesting genes. Subsequently, in plant biology, metals possess certain functions that help molecular and biosynthesis pathways to act as cofactors. For example, metals such as iron, zinc, manganese, and copper are very important and vital for organisms in this plant genus; this is because of their numerous biological actions that are essential for the host to grow [24]. Past research has revealed that iron and copper are found in high quantities in the chloroplast of plants [24]. This high content of transition metals on the plant chloroplast is because of the metalloproteins, which strictly require the interaction of these metals in the photosynthetic electron transport chain [25,26,27].

**Figure 1:**
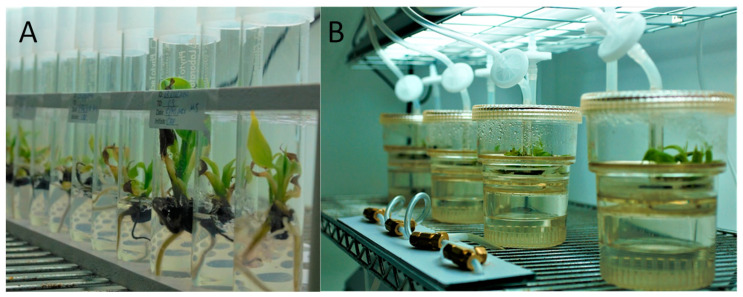
*Musa* sp. cultured in conventional micropropagation (**A**), and temporary immersion bioreactors (**B**).

Hence, we aimed to characterize the molecular, morphological, and biochemical properties of *Musa* sp. cultured with two methods (Figure 1A(CM),B(TIB)) to validate the TIB method and understand the mechanism underlying the plant enhancement. Correspondingly, to the best of our knowledge, there is no research on Rubisco small units and *PEPC* expression comparison on in vitro plantains, and no biochemical characterization that can help to elucidate what metal ions potentially interact with photosynthetic genes expression, and how this occurs. Additionally, morphological characterization using high-resolution microscopy, such as Scanning Electron Microscope (SEM), of root tip and stomata of *Musa* sp. growth on TIB and CM were unexplored. We characterized the growth of plantain in TIB at a molecular, biochemical, and morphological level to consider the potential of TIB as an alternative and cost-effective method of producing plantlets under laboratory conditions. Deciphering molecular aspects and morphological changes during in vitro culture using two methods might provide a better understanding of how this method affects plant development. This will provide a viable model for the mass production of plantain for farmers, reducing the challenges of food availability in future and the concern of disease transmission using conventional methods. The implementation of the micropropagation of plantains using bioreactors could help to establish an alternative protocol, allowing plantain to be cultured on a mass scale.

## 2. Materials and Methods

### 2.1. Plant Material

Explant selection was based in one available genotype with desirable traits (fruit size, taste, and number of produced fruits) with commercial value. Tissues were disinfected and cultured in a controlled environment. Selected tissues were propagated using the method as described in Aragón et al. [22] with some minor modifications. Conventional seeds of *Musa maricongo* were collected from a plantain farmer in Barranquitas, Puerto Rico. The collected tissue was washed with tap water and disinfected with sodium hypochlorite solution. Tissues were clipped to obtain the meristematic tissue and rinsed before being placed in a 250 mL beaker with disinfecting solution (3% Sodium hypochlorite (Clorox, Oakland, CA, USA) and 1 drop of Tween^®^ 20 (Sigma —P2287, San Luis, Misuri, USA) for overnight. Tissues were processed in the cleanroom inside a biological cabinet (Labconco Purifier Logic+ Class II, Type A2). After the first disinfection, the solution was discarded from the beakers, and the second disinfection process was carried out using 10% sodium hypochlorite solution for 30 min. Next, 3 washes were performed for approximately 2–3 min with distilled water, before the tissue was placed in an initiation media in a dark environment for 15 days at environmental temperatures ranging from 25 to 30 °C. After 15 days, the tissues were subdivided into two fragments and transplanted into propagation media (Murashige and Skoog basal salts mixtures (MS) (PhytoTechnology —M524, Lenexa, KS, USA), 3% sucrose, 13.3 µM 6-benzylaminopurine (BAP), 0.1% Murashige and Skoog vitamin solution (Sigma—M3900), and 0.25% phytagel (Sigma—P8169) for further development, with a photoperiod of 16:8, temperature varying from 25 to 30 °C, and light intensity ranging from 7976–8232 Lux (Philips - F32T8/TL965 light bulb, Andover, MA, USA). Then, the tissues were transplanted, and after 3 subcultures of ~22 days, the plantlets were ready for the first experimental trials in the TIB and CM for comparison. Finally, plantain shoots were micropropagated in a solid medium to create a stock of experimental plantlets.

### 2.2. Growth Comparison of TIB and Conventional Micropropagation

The TIB protocol and growth comparison methods were adapted from Aragon et al. [22,23], with certain modifications that included using 3 biological replicates in each of the 5 bioreactors (n = 15) and 5 sets of test tubes with 3 biological replicates (n = 15) for the CM. Different sizes and stages of development (small clusters of shoots and individual shoots) were used to identify the correct stage that tissue could be integrated into the TIB system. Each set of tubes was comparable, proportional, and similar in tissue size average (Supplementary Table S1). TIB (RITA^®^—Sigma) and CM crystal test tubes (PhytoTechnology) were used. In both methods, the basal medium was Murashige and Skoog supplemented with Plant Growth Regulator (PGR). The composition of the culture media used was MS (PhytoTechnology—M524), 3% sucrose (Sigma—S0389), 13.3 µM BAP—PGR (Phytotechnology—B130), 0.1% vitamins, and 0.25% phytagel (Sigma—P8169) (only added to the solid media). A total culture media volume of 250 mL was used in each bioreactor, while 15 mL was used in each test tube. In vitro conditions for both tested methods (TIB and CM) were as follows: temperature ranging from 25 °C to 30 °C, photoperiod of 16:8, light intensity ranging from 7976 to 8232 Lux (Philips F32T8/TL965 light bulb), and bioreactor immersion times of 3 min/4 h. Growth comparisons consisted of harvesting plants (TIB and CM) at the end of the second cycle (1 cycle = 22–25 days) for morphological and physiological analysis. At the end of the growth cycle, the following growth parameters were evaluated in plantlets cultured through both methods: growth rate in TIB (n = 15) and CM (n = 15), multiplication rate in TIB (n = 15) and CM (n = 15), microscopic imaging in TIB (n = 1) and CM (n = 1), photosynthetic activity in TIB (n = 3) and CM (n = 3), qRT-PCR in TIB (n = 3) and CM (n = 3), and ICP-OES in TIB (n = 3) and CM (n = 3).

### 2.3. RNA Isolation and cDNA Preparation

RNA extraction from plantain leaves developed in TIB (n = 3) and CM (n = 3) were performed following the protocol of Valderrama-Cháirez et al. [28] as previously modified by Rodríguez-García et al. [29]. Before converting the isolated RNA into cDNA, purification of RNA was performed using an Invitrogen DNA-free kit, following the manufacturer’s procedure as suggested for DNase treatment. cDNA was synthesized using Invitrogen Superscript VILO master mix, in which 4 µL of this master mix was added to 16 µL of each sample, as per manufacturer’s instructions. Then the samples were placed in the thermal cycler with the default settings for qRT-PCR.

### 2.4. Oligonucleotide Design

Oligonucleotide pairs for analyzing gene expression were designed using nucleotide sequences of Rubisco (*rbcS1*) (AF008214) and Actin (EF672732.1) from NCBI. The oligonucleotide pairs synthesized for the *PEPC* gene were obtained from Aragón et al. [23]. All oligos were synthesized by Sigma, Inc. (Table 1).

### 2.5. Quantitative Real-Time Polymerase Chain Reaction (qRT-PCR)

After 2 in-vitro growth cycles, cDNA from plantain leaves cultured in TIB (n = 3) and CM (n = 3) were used for qRT-PCR, with 3 replicates each. Reactions were carried out in Studio 12k Flex (Applied Biosystems, Waltham, MA, USA). Actin was used for normalizing the gene expression. The qRT-PCR reaction mixture consisted of 20 ng of cDNA, 0.1 µM gene-specific primers, and a master mix containing SBYR green. The PCR parameters were: 95 °C for 3 min, 40 cycles at 95 °C for 15 s, 59 °C for 30 s, and 72 °C for 20 s, followed by melt curve analysis. PCR products were resolved in 2% (*m*/*v*) agarose gels run at 72 V in a TBE 1× buffer together with a low molecular DNA-standard ladder to confirm the amplicon size (Supplementary Figure S1). A comparison of relative gene expression was performed using the delta–delta Ct method.

### 2.6. Photosynthetic Activity Measurement and Acclimatization

After in vitro tissue culture experiments, plantains developed on CM and TIB were successfully acclimatized under controlled conditions inside the greenhouse. In brief, ex vitro TIB plantains (n = 12) were potted in 4” plastic pots with Promix soil, with automatic irrigation under filtered sunlight for ~3 weeks. After plant acclimatization, photosynthetic activity was measured in plantains developed in TIB (n = 1, 9 observations) and CM (n = 1, 9 observations) using a JUNIOR-PAM instrument (Heinz Walz GmbH, Pfullingen, Germany). A light curve analysis was made following the manufacturer’s instructions to obtain the relative electron transport rate. Finally, crude data of the Electron Transport chain Rate (ETR) (µMol/m^2^/s) and Photosynthetically Active Radiation (PAR) (µMol/m^2^/s) were plotted in excel (ETR on Y-axis and PAR in X-axis) to construct a graph to visualize the data obtained from the analysis; additionally, a Student’s *t*-test was conducted at 95% confidence level to verify the statistical significance.

### 2.7. ICP-OES Qualitative Analysis

Qualitative metal analysis was applied using ICP-OES (Shimadzu ICPE-9820, USA). In brief, fresh plantain leaf tissue after two growth cycles of an average plant size of 4.32 cm (CM) and 8.01 cm (TIB) were taken from the experimental plants cultured in CM (n = 3) and TIB (n = 3) for elemental composition analysis, with 3 technical replicates each for a total of n = 9 for both CM and TIB. Approximately 0.5 g of tissue was weighed. Tissue was digested in 10 mL of 2% nitric acid solution. Then, samples were placed in the microwave digestion and extraction system (CEM Mars 6). Digested samples were passed to ICP-OES and parameters were adjusted for the injections of the samples and measurement of the elemental distribution and composition.

### 2.8. Scanning Electron Microscopy

Fresh leaf and root tissues of plantains in the propagation stage and cultured for 1 growth cycle (~46 days) were used for morphological analysis of stomata and root tips. Samples from TIB (n = 1), and CM (n = 1) were placed in fixing solution (2% Formaldehyde in PBS buffer) for 1 h at room temperature, followed by two washes in 1× PBS (15 min), and distilled water (15 min). Then, samples were dehydrated by low–high gradients of ethanol (25%, 50%, 75%, 85%, 95%, 100%), and samples were passed through each ethanol solution for 15 min. Afterward, each sample was attached to a carbon sticker in a metal stub. Samples were dried for 30 min and gold coated to a 7 nm thickness in Luxur Gold coater. Roots and leaves were analyzed under Scanning Electron Microscope (Thermo Scientific Phenom Pro X) to determine potential morphological differences between plantain organs developed in TIB and CM.

### 2.9. Statistical Analysis

Paired Student’s *t*-test (95% confidence) was applied to verify the statistical significance of the morphological, biochemical, and molecular characteristics of plantain growth in TIB and CM.

## 3. Results

### 3.1. Comparison of Growth Parameters in TIB vs. Conventional Micropropagation

**Figure 2:**
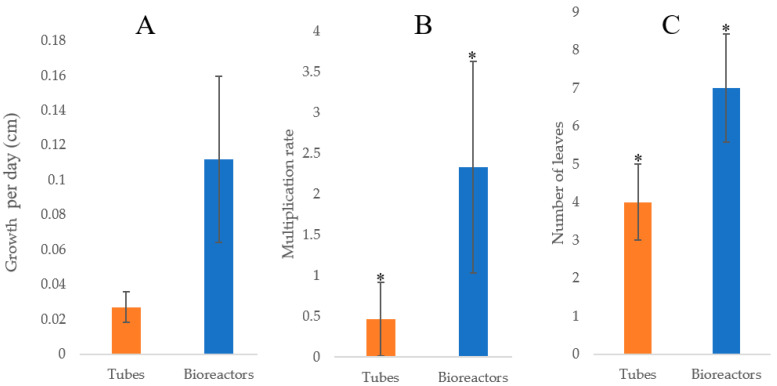
In vitro plantain development comparison of CM vs. TIB methods: (**A**) Growth rate, (**B**) multiplication rate, and (**C**) number of leaves per plantlet. Asterisk (*) indicates significant difference determined by Student *t*-test at 95% confidence level, (n = 15).

**Figure 3:**
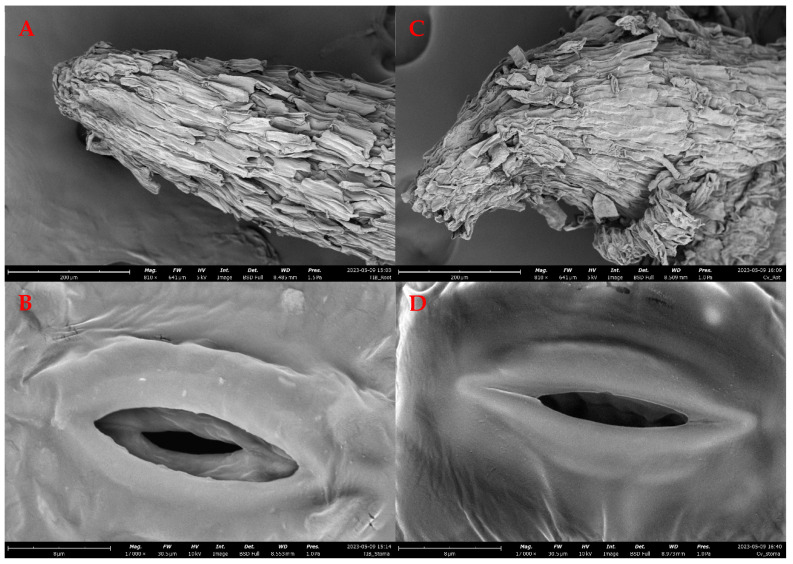
SEM images of morphological characteristics of in vitro plantains. TIB plantain root (**A**) and stomata (**B**). CM plantain root (**C**) and stomata (**D**).

**Figure 4:**
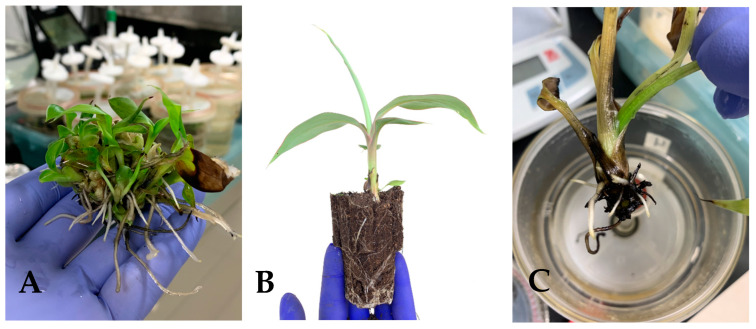
(**A**) Shoot clusters of plantain multiplication in TIB; (**B**) healthy plantain seedling and (**C**) root formation after 20 days.

The regenerants were evaluated in vitro using two different methodologies: TIB and CM. The morphological characteristics and data obtained exhibited morphological differences between the tissues grown in TIB as compared to CM. Furthermore, the growth rate (Figure 2A), multiplication rate (Figure 2B), and total leaves per plantlet (Figure 2C) of the plantains showed a higher development in TIB. Statistical results suggest that there was no significant difference (*p* = 7.9309) in the growth rate, but there was a significant difference in the multiplication rate (*p* = 0.01676) and the total leaves per plantlet (*p* = 0.0017) of plantains cultured in TIB. SEM topography images showed differences only in the subterranean system, specifically the root tip (Figure 3A–C). In contrast, the aerial part did not show any morphological differences (Figure 3B–D). Additionally, mass propagation of shoot clusters was seen only in plantain growth in TIB (Figure 4A). The first plantain developed in TIB was successfully fully acclimatized (Figure 4B). Complex root formation occurred at day 20 in TIB-grown tissue (Figure 4C).

### 3.2. Gene Expression

**Figure 5:**
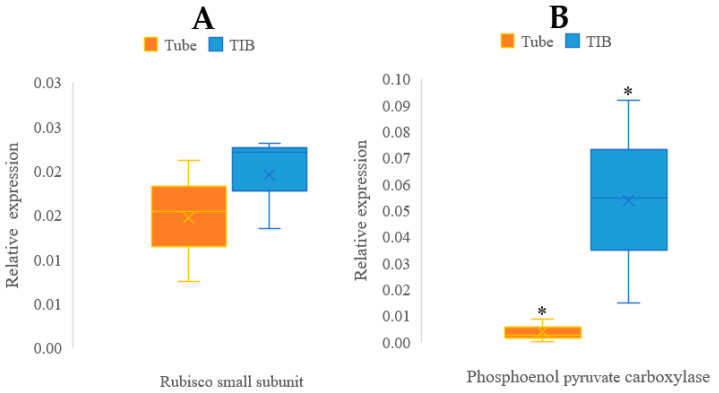
Relative gene expression of photosynthetic genes. *rbcS1* (**A**); *PEPC* (**B**); comparison between *Musa* sp. growth in TIB and CM. Asterisk (*) indicates significative difference determined by Student’s *t*-test at 95% confidence level, (n = 9).

qRT-PCR data obtained showed a high transcript abundance of both *PEPC* and *rbcS1* genes in plantains cultivated in TIB compared to the ones grown in CM (Figure 5). Statistical analysis using Student’s *t*-test (95% confidence) suggested a significant difference in *PEPC* transcript abundances between TIB and CM cultivated tissues (*p* = 0.0447), while Rubisco small subunits showed no statistical difference (*p* = 0.1936) in transcript abundance between the culture methods.

### 3.3. Photosynthetic Activity

**Figure 6:**
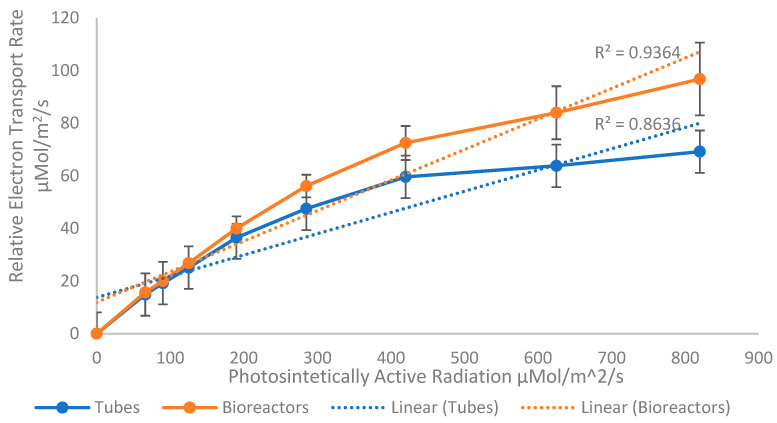
Photosynthetic activity comparison between acclimatized *Musa* sp. growth in TIB and CM. No statistical difference was determine using Student’s *t*-test at 95% confidence level, (n = 1, 9 observation).

After ~3 weeks of the plants being fully acclimatized under controlled conditions in the greenhouse, plantains cultivated in TIB showed a higher relative electron transport chain rate in photosynthetically active pathways compared to the ones cultivated in CM (Figure 6). However, these data, analyzed using the Student’s *t*-test, demonstrated that there were no significant differences (*p* = 0.5476) in the photosynthetic activity between the plants grown in variable growth settings. 

### 3.4. Digestion and ICP-OES Qualitative Analysis

**Figure 7:**
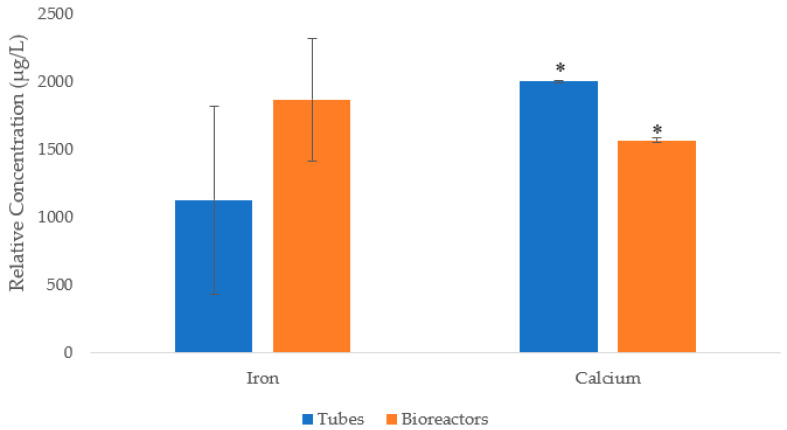
Comparison of the transition metals present in *Musa* sp. growth in vitro. Asterisk (*) indicates significative difference determined by Student’s *t*-test at 95% confidence level, (n = 9).

A comparative qualitative assay detected the presence of all metals in plantain tissues (Supplementary Figure S2; Table S2). However, the relative concentration of the detected transition metals showed a higher presence of Iron (Fe) in plants growing in TIB than in the ones grown in the CM. However, a statistical test was applied to verify the significant difference in Fe content between the methods. Statistical analysis (Student’s *t*-test) showed no statistical differences (*p* = 0.2736) between the Fe content in plantains grown in TIB and CM. Moreover, Fe was present in higher quantities in comparison with the other elements of interest studied (Figure 7). However, the Student’s *t*-test showed a significant difference in calcium content (*p* = 0.0226).

## 4. Discussion

Plantain growth comparison between the CM and TIB methods demonstrated better morphological characteristics in TIB. Shoot elongation, length of roots, multiplication rate, and growth rate were similar to previously documented reports [23,24,25,26,27,28,29,30]. These results demonstrate efficiency of TIB as it provides numerous conditions for optimum development of plant tissue, such as oxygen supply/transport, space, nutrient acquisition, and immersion ranges [11,12]. We saw during the experimental procedure that the headspace, air supply, and temporary immersion of liquid media (3 min/4 h) with a photoperiod of 16/8 h improved plants’ health and development [22], thus showing formation of a complex root at day 20 in the TIB system. Additionally, SEM images highlighted morphological differences in the root structures between plantains grown in TIB and those grown in CM. Tropism could play a key role in the difference in root development; according to Izzo & Aronne [31], tropism controls organ positions during plant development. Specifically, TIB-grown plantains show healthy and elongated root tips, while in CM they show non-elongated and curved root tip development. Curved root elongation in CM plantains might be associated with physiochemical properties of the culture media and in vitro methodology. Roots can interact with the surrounding environment and can reorganize and redirect their growth away or in the direction of physiochemical signals, such as temperature, oxygen, gravity, light, water, and chemicals [32]. Roots developed in CM can potentially suffer from lack of oxygen. Previous studies have reported better root and other organ development in TIB as compared to CM [23], as roots might move towards the surface in CM. By contrast, TIB plantain roots proved to be healthy and elongated, which perhaps can be associated with the microenvironment inside TIB, which mimics outdoor conditions and promotes optimal plant development [23]. Additionally, stomatal structures showed high similarity in plantain growth in both methods, suggesting limited effects in stomatal morphology. Scanning stomata at various developmental stages could provide an insight into the effect of growth conditions in the morphology of stomata. Additionally, air supply increased the interchanges of gases in the headspace of the bioreactors, helping to simulate a natural environment, thus helping ensure the plants experienced minimal stress during in vitro and ex vitro acclimatization [23,24,25,26,27,28,29,30,31,32,33,34]. Furthermore, the interchange of gases is linked to PEPC, an enzyme that is involved in carbon assimilation on photosynthetic pathways [21]. Uma et al. [30] demonstrated a 2.7-fold increase in tissue multiplication on TIB in comparison with CM. Growth comparison (growth rate, multiplication rate, number of leaves), morphological characterization (SEM images), and biochemical and molecular contrast (ICP-OES, qRT-PCR, and photosynthetic activity) suggested an improvement in plantain multiplication rate and an overall enhancement of development in plantlets using the TIB bioreactors as a culture system. In brief, we provide data that support the enrichment and upscaling of plantain propagation using TIB over CM.

In addition, molecular analysis was conducted to study representative photosynthetic gene expression during in vitro development of plantains under two different methodologies. Results showed no significant difference in *rbcS1* transcripts abundance. However, the region of Rubisco studied is reported for the first time in our study on TIB plantains. The region studied was the small subunit related to producing conformational changes in the other subunits, promoting the catalytic rate [19]. There was significant upregulation of the *PEPC* gene in plantains cultivated in TIB due to the conditions inside the bioreactor (air supply and gas interchanges) which might have influenced the expression of *PEPC* [22]. The PEPC gene shows a significantly high transcript abundance in plantains cultivated in TIB compared to CM. High efficacy of carbon assimilation in plants inside TIB may occur [23]; however, carbon assimilation can be influenced by other factors, such as the action of 3-phosphoglycerate kinase and pyruvate kinase, which should be considered in further studies to validate our findings. The carbon concentration produced by the PEPC pathway controls the process of photorespiration, thus promoting the efficiency of photosynthesis [35] under TIB conditions. Additionally, as mentioned previously, the PEPC enzyme is related to the interchange of gases in plant tissue, specifically in leaves, and when this enzyme is upregulated, the rates of stomata opening increase significantly, suggesting that the interchange of gases between the plant and TIB is effective and promotes plant organ differentiation/multiplication. The high transcript abundance of *PEPC* in TIB induced plantlets may be related to the healthy and vigorous growth of these plantlets [23]. This suggests that plantains produced in TIB may be photosynthetically more active than the ones produced in CM. Additionally, comparative electron transport rate measurements of fully acclimatized plantains obtained from TIB and CM over ~3 weeks suggest that TIB plantains continue to possess high photosynthetic activity weeks after their acclimatization. Although no statistical difference in photosynthetic rate was observed in plantains grown in TIB and CM in ex vitro conditions, further comparative growth analysis during in-vitro and ex-vitro conditions needs to be applied. However, previous data show a relation between light intensity and gases such as CO_2_ and photosynthetic rate during in vitro plant development and PEPC activation [21]. This corroborates previously documented data, which explains the presence [36,37] and essential [38] role of gas interchanges in TIB headspace for improved growth development.

The elemental composition of plantains in TIB was analyzed to study the presence of certain metals of interest that potentially interact with the photosynthesis pathway. All metals of the periodic table were tested for in plantains grown in CM and TIB. Substantial differences in some elements of interest, including boron, calcium, and the transition metals iron (Fe), manganese (Mg), zinc (Zn), and copper (Cu) were obtained, and might provide an explanation for the difference in plant development in CM and TIB. Despite the fact that there was no statistical difference between CM and TIB in iron content, iron was present in greater quantity than all other elements of interest, and was relatively high in TIB. This was expected, since previous data [24] suggested that copper, zinc, manganese, and specifically, iron acted as cofactors in some metabolic pathways in plants. These features suggest that Fe might be associated with the high transcript abundance of PEPC in TIB plantains and may be involved with PEPC proteins. Additionally, it is possible that Fe may be involved in the chloroplast, where carbon metabolism occurs. Most of the pathways that occur in this organelle require the presence of metals as cofactors [24]. It is known that Fe acts as a cofactor of some photosynthetic pathways [24], and interaction between iron and PEPC may occur during the activation/signaling of transcription factors related to these two photosynthetic genes. Previous research [25] revealed that iron and copper are present in high quantities in chloroplast, and the high contents of these metals are linked to metalloproteins that strictly require these cofactors for the photosynthetic electron transport chain. We tested the relative electron transport rate in acclimatized plantains developed in CM and TIB, and the results supported previous data, which explained the crucial and important roles these metals play as cofactors and regulators in photosynthetic pathways [25,26,27,28,29,30,31,32,33,34,35,36,37,38,39]. Transition metals are essential for various metabolic paths that are crucial for healthy plant development [20]. One potential reason why plantains produced in bioreactors possess an enhanced phenotype is the presence of these metals, providing biotic and abiotic stress resistance during ex vitro conditions [39,40]. In addition, other important elements, such as copper and calcium, show a higher relative concentration in leaf tissues of plantains grown in CM in comparison to the TIB. Differences in plantain pseudostem development between TIB and CM may be an effect of calcium concentrations, because of the essential role of calcium in cell wall synthesis and structure [41]. We suggest that because CM plantains are compressed in a small environment, transport elements such as Ca contribute to the development of a rigid cell wall. The lower relative concentration of Ca in TIB plantains suggest an explanation as to why TIB plantains show a dynamic or flexible pseudostem development.

However, quantitative elemental analysis should be addressed to reveal more specific quantification of elemental composition of plantain tissues. Additionally, histology and histochemical analysis of plantain cell wall tissues should be addressed to localize and validate the presence of key photosynthetic/carbon assimilation enzymes during development in TIB and CM. In brief, our data suggest that morphological, biochemical, and molecular analysis may help to understand how plants develop in vitro and how the method of cultures can be improved for producing high-quality plants. Additionally, to the best of our knowledge, we report here the first plantain plantlet developed through a RITA^®^ bioreactor system in Puerto Rico.

## 5. Conclusions

Evaluation of both in vitro methods (liquid medium in TIB and solid media in CM) demonstrates an improvement in plant development, growth, and multiplication rate in the TIB system compared to CM. Morphological and imaging aspects need validation through molecular techniques to further validate the functions. Photosynthetic gene expression analysis shows high transcript abundance of *PEPC* in plants cultured in TIB. Additionally, to the best of our knowledge, Rubisco small subunits (*rbcS1*) and *PEPC* expression were tested for the first time in TIB in this study. The photosynthetic activity measurement using the Junior PAM system helped to validate the gene expression analysis, giving an overview of this activity after plant acclimatization. These results suggest experimental genes can still be upregulated during the acclimatization of plants obtained from TIB. Finally, we conclude that TIB is a viable tool for increasing plantain propagation, health, and strength more rapidly than CM. These results demonstrate that micropropagation of *Musa* spp. via the liquid medium in TIB is an efficient and low-cost approach in comparison to the conventional solid media in CM.

## Figures and Tables

**Table 1:** Quantitative PCR *Musa* sp. Oligonucleotide Sequences.

Genes	Amplicon Size	Sequences	NCBI Accession Number
*Actin*	121 bp	5″AAGTACAGTGTCTGGATTGG 3″ 3″GTTTCGCTGCTATTTCATGA 5″	EF672732.1
*rbcS1*	73 bp	5″ TGTTTTTGTTTCCCCAAGAA 3″ 3″ ATGGACGTTTCGCTTTATTTA 5″	AF008214
*PEPC*	223 bp	5″ GGTAGTGGAAATGTCTCGCTTGG 3″ 3″ GCAATCCATGAACCTGAGAAGCC 5″	Z99987.1

## Data Availability

Not applicable.

## Supplementary Materials

The following supporting information can be downloaded at: https://www.mdpi.com/article/10.3390/plants12213770/s1. Figure S1. Title: Melting Curves and Electrophoresis results; Figure S2. Title: ICP-OES element composition; Table S1: Plants size for experimental trial; Table S2: ICP–OES Semi-quantitative data.
