# Supplementary material for: Biochemical and Molecular Characterization of Musa sp. Cultured in Temporary Immersion Bioreactor
Source: Plants (Basel). 2023 Nov 4;12(21):3770. doi: 10.3390/plants12213770 (PMC10647254; doi:10.3390/plants12213770)
Supplement: Supplementary file 1 [file plants-12-03770-s001.zip › plants-2598406-supplementary.pdf]

Supplementary material

Supplementary table 1. Plants size for experimental trial.

Tubes	Size (cm) Initial	Size (cm) Final	Bioreactor Samples	Size (cm)	Size (cm) Final
M1	2.2	2.4	M1	3.0	7
M1	2.0	5.0	M1	1.9	5.6
M1	1.6	2.0	M1	2.5	7.4
M2	2.4	4.8	M2	3.5	6.4
M2	2.3	4.1	M2	2.4	6.7
M2	3.4	4.5	M2	2.0	10.5
M3	3.9	4.6	M3	2.5	4.1
M3	2.0	2.8	M3	2.3	4.0
M3	2.4	3.8	M3	2.4	2.5
M4	2.9	3.7	M4	3.1	8.9
M4	3.2	3.8	M4	3.6	10.6
M4	2.7	4.2	M4	3.9	11.5
M5	8.8	9.3	M5	6.3	11.9
M5	4.1	4.2	M5	5.4	12.5
M5	4.2	5.6	M5	4.6	10.6

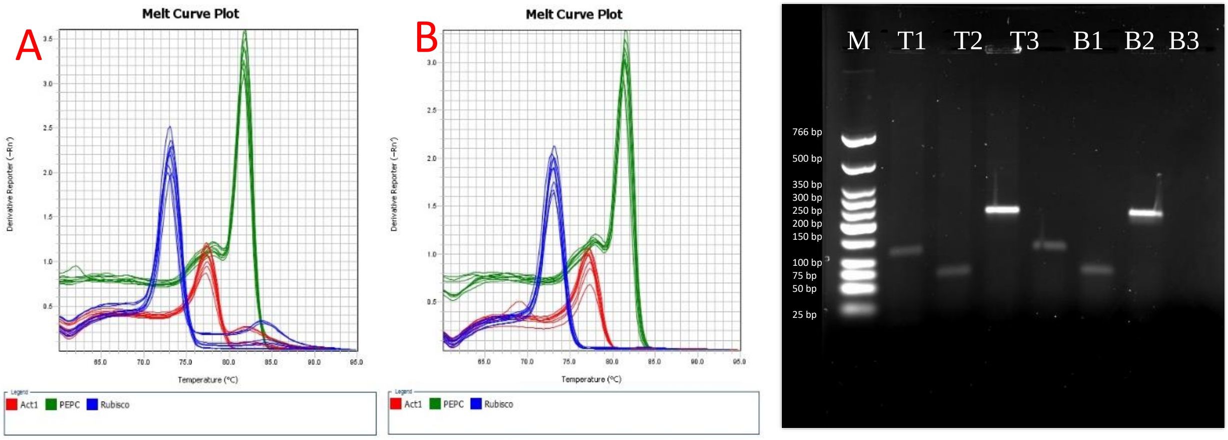

Supplementary figure 1. Melting curve of plantains samples from TIB (A) and CM (B). Electrophoresis on 2% agarose gel result from qPCR. (M) = Low molecular marker. 1-3 = samples from tubes, 4-6 = samples from bioreactors. The expected amplicon for the 3 genes of interested are visible on the gel.

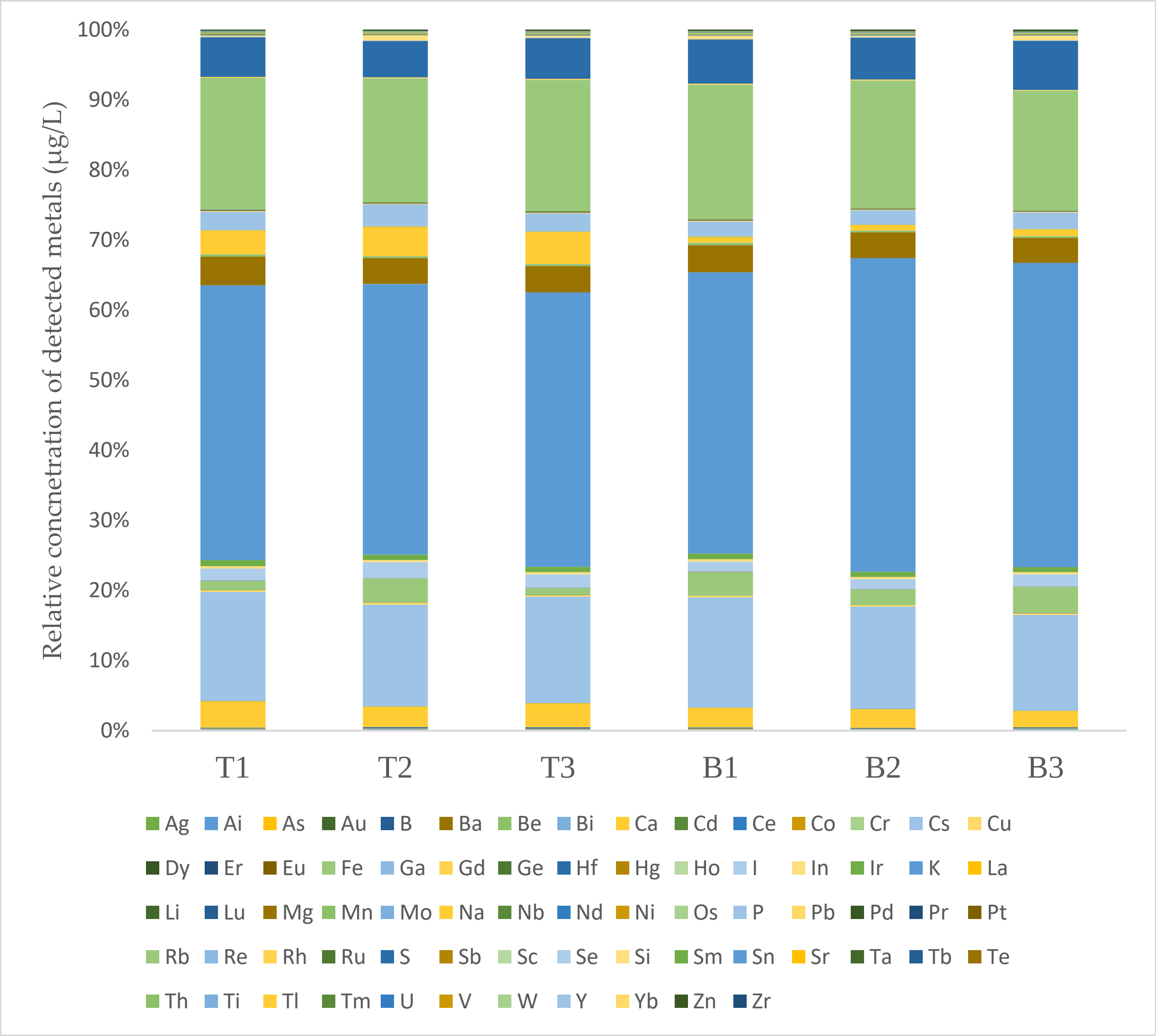

Supplementary figure 2: Elemental composition of conventional micropropagation and temporary immersion bioreactors. [n=3].

Supplementary table 2. ICP – OES Semi-quantitative data

Metals	T1 ug/L	T2 ug/L	T3 ug/L	**Avg Relative concentration ug/L**	B1 ug/L	B2 ug/L	B3 ug/L	**Avg Relative concentration ug/L**
Ag	13	12	12	**12.33**	13	12	12	**12.33**
Ai	120	160	120	**133.33**	110	110	180	**133.33**
As	44	42	42	**42.67**	42	41	41	**41.33**
Au	5.8	5.8	5.5	**5.70**	5.8	5.5	5.6	**5.63**
B	64	93	90	**82.33**	73	59	64	**65.33**
Ba	1.2	1.3	1.3	**1.27**	0.72	0.99	0.88	**0.86**
Be	0.72	0.73	0.74	**0.73**	0.75	0.75	0.76	**0.75**
Bi	24	23	23	**23.33**	23	22	22	**22.33**
Ca	2200	1800	2000	**2000.00**	1600	1600	1500	**1566.67**
Cd	2.3	2.2	2.2	**2.23**	2.2	2.1	2.2	**2.17**
Ce	12	12	12	**12.00**	12	12	12	**12.00**
Co	8.2	8.1	7.8	**8.03**	8.1	7.8	7.9	**7.93**
Cr	10	8.1	7.8	**8.63**	8	7.7	7.9	**7.87**
Cs	9100	9000	8900	**9000.00**	9000	8800	8800	**8866.67**
Cu	150	190	140	**160.00**	130	130	150	**136.67**
Dy	1.9	1.9	1.9	**1.90**	1.9	1.8	1.8	**1.83**
Er	2	4.5	3.5	**3.33**	3.7	2.8	4.2	**3.57**
Eu	1.1	1	1	**1.03**	1	1	1	**1.00**
Fe	730	2100	550	**1126.67**	1900	1300	2400	**1866.67**
Ga	26	25	25	**25.33**	25	24	25	**24.67**
Gd	3.3	3.1	3.1	**3.17**	3.1	3.1	3.1	**3.10**
Ge	14	14	14	**14.00**	14	13	13	**13.33**
Hf	25	25	24	**24.67**	25	24	24	**24.33**
Hg	2	1.9	1.9	**1.93**	2	1.8	1.9	**1.90**
Ho	4.4	4.3	4.2	**4.30**	4.3	4.2	4.2	**4.23**
I	1000	1400	1100	**1166.67**	820	850	1100	**923.33**
In	190	190	190	**190.00**	190	190	190	**190.00**
Ir	500	470	450	**473.33**	470	450	470	**463.33**
K	23000	24000	23000	**23333.33**	23000	27000	28000	**26000.00**
La	4.6	4.5	4.5	**4.53**	4.5	4.4	4.4	**4.43**
Li	1.2	1.1	1.1	**1.13**	1.1	1	1.1	**1.07**
Lu	1.5	1.5	1.4	**1.47**	1.5	1.4	1.4	**1.43**
Mg	2400	2300	2200	**2300.00**	2200	2200	2300	**2233.33**
Mn	160	140	150	**150.00**	180	150	140	**156.67**
Mo	19	19	19	**19.00**	19	18	20	**19.00**
Na	2000	2600	2700	**2433.33**	470	470	610	**516.67**
Nb	3.9	3.9	3.8	**3.87**	3.9	3.7	3.8	**3.80**
Nd	14	13	13	**13.33**	13	13	13	**13.00**
Ni	19	25	8.4	**17.47**	16	8.2	17	**13.73**
Os	37	37	35	**36.33**	37	35	36	**36.00**
P	1500	1900	1500	**1633.33**	1200	1200	1500	**1300.00**
Pb	70	70	67	**69.00**	70	66	69	**68.33**
Pd	64	62	60	**62.00**	61	59	60	**60.00**

Pr	14	13	13	**13.33**	13	13	13	**13.00**
Pt	34	33	33	**33.33**	33	32	32	**32.33**
Rb	11000	11000	11000	**11000.00**	11000	11000	11000	**11000.00**
Re	11	11	11	**11.00**	11	10	11	**10.67**
Rh	77	76	75	**76.00**	76	74	74	**74.67**
Ru	15	16	15	**15.33**	16	15	15	**15.33**
S	3300	3200	3400	**3300.00**	3600	3600	4500	**3900.00**
Sb	27	26	26	**26.33**	26	25	26	**25.67**
Sc	0.61	0.59	0.59	**0.60**	0.59	0.58	0.58	**0.58**
Se	48	47	46	**47.00**	47	45	46	**46.00**
Si	69	420	140	**209.67**	220	98	420	**246.00**
Sm	8.1	7.8	7.8	**7.90**	7.9	7.6	7.6	**7.70**
Sn	53	52	52	**52.33**	53	50	52	**51.67**
Sr	1	0.91	0.98	**0.96**	0.65	0.72	0.9	**0.76**
Ta	16	16	15	**15.67**	16	15	16	**15.67**
Tb	8.4	8.2	8.2	**8.27**	8.3	8.1	8.1	**8.17**
Te	48	47	45	**46.67**	45	44	45	**44.67**
Th	86	85	82	**84.33**	85	82	83	**83.33**
Ti	1.1	2.8	2.2	**2.03**	2.3	1.7	2.6	**2.20**
Tl	50	49	48	**49.00**	49	46	48	**47.67**
Tm	4.1	4.1	4	**4.07**	4.1	4	4	**4.03**
U	64	62	62	**62.67**	62	61	60	**61.00**
V	3	2.9	2.9	**2.93**	2.9	2.9	2.9	**2.90**
W	48	47	46	**47.00**	47	45	46	**46.00**
Y	0.53	0.51	0.51	**0.52**	0.51	0.5	0.5	**0.50**
Yb	0.4	0.39	0.38	**0.39**	0.38	0.37	0.38	**0.38**
Zn	110	130	130	**123.33**	130	150	170	**150.00**
Zr	1.8	1.8	1.8	**1.80**	1.8	1.7	1.7	**1.73**
